# Biochemical and Functional Interactions of Human Papillomavirus Proteins with Polycomb Group Proteins

**DOI:** 10.3390/v5051231

**Published:** 2013-05-14

**Authors:** Margaret E. McLaughlin-Drubin, Karl Munger

**Affiliations:** Division of Infectious Diseases, Brigham and Women’s Hospital and Department of Medicine, Harvard Medical School, 181 Longwood Avenue, MCP 861, Boston, MA 02115, USA

**Keywords:** cervical cancer, biomarker, histone methylation, tumor suppressor

## Abstract

The role of enzymes involved in polycomb repression of gene transcription has been studied extensively in human cancer. Polycomb repressive complexes mediate oncogene-induced senescence, a principal innate cell-intrinsic tumor suppressor pathway that thwarts expansion of cells that have suffered oncogenic hits. Infections with human cancer viruses including human papillomaviruses (HPVs) and Epstein-Barr virus can trigger oncogene-induced senescence, and the viruses have evolved strategies to abrogate this response in order to establish an infection and reprogram their host cells to establish a long-term persistent infection. As a consequence of inhibiting polycomb repression and evading oncogene induced-senescence, HPV infected cells have an altered epigenetic program as evidenced by aberrant homeobox gene expression. Similar alterations are frequently observed in non-virus associated human cancers and may be harnessed for diagnosis and therapy.

## 1. Introduction

### 1.1. Human Papillomaviruses

Human papillomaviruses (HPVs) are members of the *Papillomaviridae,* a large family of viruses that infect epithelial cells. Papillomaviruses consist of approximately 8 kb double stranded circular DNA genomes packaged into 55 nm icosahedral capsids. Only one of the two strands is transcribed and encodes five to six “early” (E) open reading frames (ORFs) that encode non-structural regulatory proteins and two “late” (L) ORFs, L1 and L2, from which the major and minor capsid proteins, respectively, are translated. Papillomaviruses exhibit exquisite species specificity, and as a consequence there are no heterologous animal systems to model the natural course of HPV infections. 

Almost 200 HPV genotypes have been identified, and they are categorized by the degree of sequence similarity into specific papillomavirus genera [1]. The alpha HPVs have received considerable experimental attention, since they are classified as “high-risk” and “low-risk” based on the propensity for malignant progression of the lesions that they cause. Low-risk HPVs such as HPV6 and HPV11 cause generally benign warts of the genital or oral mucosa whereas infections with “high-risk” HPVs cause lesions of these same target tissues that can undergo malignant progression [2]. Almost 100% of human cervical cancers as well as a large fraction of other anogenital tract and oral carcinomas are caused by high-risk alpha HPV infections [3]. Even though highly efficacious prophylactic vaccines—that prevent infection with the most highly abundant low-risk HPVs (HPV6 and HPV11) and high-risk HPVs (HPV16 and HPV18)—are on the market, the acceptance of these vaccines in the US population has been low [4]. Moreover, because of their cost, these vaccines are not universally available to populations in low-income countries, where cervical carcinoma remains one of the leading causes of cancer death in women [5]. In addition, cervical carcinoma generally develop years or decades after the initial infection and since the prophylactic vaccines do not protect from pre-existing infections or prevent malignant progression, it will be decades before these vaccines will have a noticeable impact on the incidence of HPV-associated cancers [6]. Hence, infections with high-risk alpha HPVs will continue to be one of the most common sexually transmitted diseases and a major cause of morbidity and mortality for decades to come.

**Figure 1:**
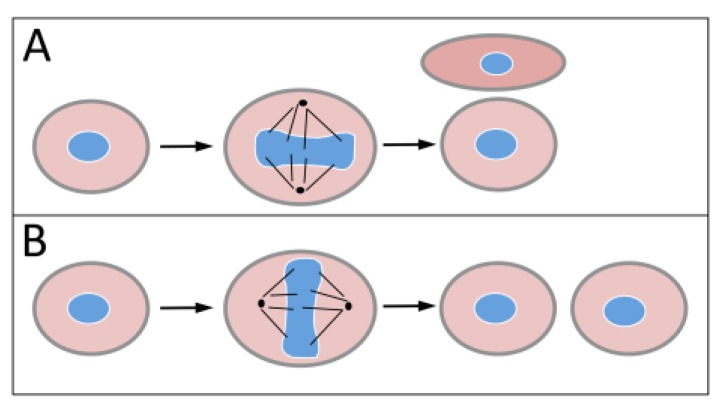
Cell division in a squamous epithelium. (**A**) Asymmetric cell division of a basal epithelial cell yields two unequal daughter cells. One remains an undifferentiated, DNA synthesis competent basal epithelial cell whereas the other daughter is a differentiating epithelial cell that will terminally withdraw from the cell division cycle. Human papillomaviruses (HPV) genomes segregated into such cells will retain these cells in a DNA replication competent state and complete their full replicative life cycle including viral progeny synthesis. (**B**) Symmetric cell division will yield two basal like cells. This mode of cell division is to replenish basal epithelial cell during wound healing. After the initial infection, HPVs establish a long-term persistent infection of these cells where the viral genome is maintained at a low copy number. The viral genome undergoes replication every time the cell replicates its genome. See text for details.

Like other viruses, HPVs are obligatory intracellular parasites that need to reprogram the infected host cell to establish an infection and complete their life cycles and produce viral progeny. In the case of HPVs that is a particularly challenging endeavor. First of all, despite extensive splicing due to their limited coding potential, HPVs can only produce a limited number of non-structural proteins. Only two of these, E1 and E2, are mechanistically involved in viral genome replication. E1 has ATPase and DNA helicase activity, and in complex with E2, it binds the viral origin of replication, where it assembles into a hexameric complex that forms a scaffold for binding cellular replication proteins [7]. HPVs do not encode any other proteins that are rate-limiting for replication such as nucleotide biosynthesis enzymes or a DNA polymerase, and these viruses are acutely dependent on the availability of the cellular DNA synthesis machinery to replicate their genomes. Moreover, the productive HPV life cycle is coupled to the differentiation status of the infected squamous epithelial cell [8]. The squamous epithelium is the largest organ of the human body and is in a constant state of self-renewal. Within this tissue, basal epithelial cells are the only cells that undergo cell division and are the targets of HPV infection. To maintain the integrity of the multilayer epithelial structure, basal epithelial cells divide asymmetrically. One daughter cell retains undifferentiated, DNA synthesis-competent basal cell characteristics, whereas the other daughter cell is poised to undergo cell cycle withdrawal and terminal differentiation, while it is pushed towards the surface of the epithelium and is eventually sloughed off. When the epithelium is injured, however, some basal cells can also undergo symmetrical division to replenish the population of basal cells and close the wound (Figure 1). Since the productive HPV life cycle including production of viral progeny only occurs in terminally differentiated cells, the virus needs to encode proteins that uncouple cell cycle withdrawal from the differentiation program to allow differentiating cells to remain DNA synthesis competent so as to permit viral genome replication. The viral E6 and E7 proteins, which in high-risk HPVs have potent oncogenic activities, are the major drivers of this process. A second and equally important aspect of the viral life cycle is the ability of high-risk HPVs to establish a long-term persistent infection of undifferentiated, basal epithelial cells [8]. One attractive model is that during persistent infection, cells harboring HPV genomes either undergo division only infrequently or that they preferentially undergo symmetrical cell division, yielding two equal basal like cells where the genome is maintained but no infectious progeny is produced. This model has not been tested experimentally, mostly because there are no adequate experimental systems that allow long-term study of a squamous epithelium *in vitro*. 

In high-risk HPV-associated cancers the viral genome frequently integrates during malignant progression and only two viral genes, E6 and E7 are consistently expressed in cervical carcinoma cells [9,10]. E6 and E7 each have potent transforming activities and can immortalize primary human epithelial cells and induce genomic instability that drives development of fully transformed, carcinogenic cells [11]. Expression of E6 and E7 in transgenic mice from a keratin K14 promoter that drives their expression in basal epithelial cells in combination with low dose estrogen treatment causes cervical carcinoma development [12]. Cervical carcinoma cells are “addicted” to E6/E7 expression and undergo cell cycle arrest, senescence or cell death when E6/E7 expression is extinguished [13,14,15]. Hence, HPV E6/E7-mediated cellular reprogramming for the purpose of establishing and maintaining a long-term persistent infection and to generate viral progeny is a risky proposition for the infected host cell as well as the virus, as it can lead to cancer formation and the ultimate demise of the host and the viral genome that is trapped within that host.

In addition to targeting specific transcriptional programs, HPV E6 and E7 proteins are known to affect epigenetic control mechanisms to more globally perturb the transcriptional competence of the infected host cells. Not surprisingly, high-risk HPV E6 and E7 proteins have been reported to biochemically interact with and/or functionally alter a variety of epigenetic enzymes including DNA methylases and histone modifying enzymes. This article focuses on interactions of the HPV E7 protein with components and regulators of Polycomb group (PcG) proteins.

### 1.2. Polycomb Group Proteins and Homeobox Genes

PcG genes were discovered in *Drosophila melanogaster* and named to denote a mutant phenotype that, in addition to segmentation defects, displays formation of aberrant sex combs on the legs of male flies. PcG proteins form macromolecular repressor complexes, polycomb repressive complexes (PRCs), that globally regulate transcriptional competence of genes that play important roles in cell fate specification and maintenance of stem cell pools [16].

**Figure 2:**
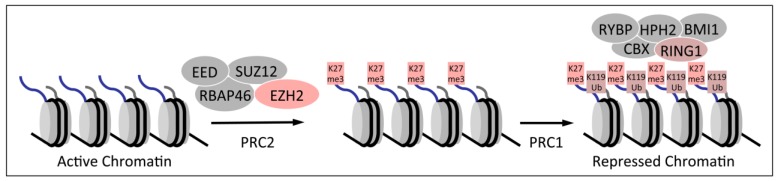
Polycomb mediated silencing of gene expression. The polycomb repressive complex 2 (PRC2) silences chromatin by trimethylating lysine residue 27 (K27me3) of histone H3, causing chromatin compaction. H3K27me3 marked chromatin is bound by PRC1, which further silences gene expression by monoubiquitinating lysine residue 119 (K119Ub) of histone H2A.

There are two major PRC species, designated PRC1 and PRC2, which play critical roles in epigenetic gene silencing. PRC2 contains the EZH2 enzyme, which places a trimethyl mark on lysine 27 of histone H3 (H3K27me3). This repressive mark causes chromatin compaction and gene silencing. PRC1 occupies H3K27me3 marked chromatin and further silences the chromatin by mono-ubiquitination of lysine 119 on histone H2A (H2AK119Ub) (Figure 2). Some PRC1 complexes can also silence gene expression in the absence of repressive H3K27me3 marks since H2AK119Ub marked chromatin is a binding site for the L3MBTL2 protein, which then establish repressive structures [17] that are particularly important in pluripotent stem cells [18].

There is compelling evidence that PRCs regulate epithelial cell differentiation as well as expansion of basal cell pools during wound healing [19,20,21]. As described in more detail above, HPVs may need to target both of these processes to allow for viral progeny synthesis and establishment of long-term persistent infection, respectively. Hence, PRC components and molecules that regulate their activity are attractive targets for HPV proteins.

The best-known transcriptional targets of PRCs are the homeobox (*HOX*) genes, which encode a family of related transcription factors that contain a homeodomain. *HOX* family members are frequently dysregulated during carcinogenesis [22,23] and germline *HOXB13* mutations have been linked to an increased risk for the development of prostate cancer [24]. HOX proteins are not only downstream transcriptional targets of PRCs, but they also mediate PRC repressor activities on some of their target genes [25].

## 2. Dysregulation of Homeobox Gene Expression in Cervical Cancers

Not surprisingly, dysregulated HOX gene expression has also been reported during cervical carcinogenesis. In an early study, *HOX* gene expression was analyzed in a series of cervical carcinoma lines and compared to expression in normal cervical epithelium. These studies documented aberrant expression of *HOXA1*, *B2*, *B4*, *C5*, *C10* and *D13* in some of the cancer lines but there was no evidence for coding mutations in *HOX* genes [26]. Differential *HOXC5* expression was also observed in a study where *HOX* gene expression in normal keratinocytes was compared to the HPV16 positive SiHa cervical carcinoma line. This study also provided evidence for increased expression of *HOXC8* in SiHa cells [27], and another study provided additional evidence for *HOXB4* overexpression in cervical carcinomas [28]. Collectively, however, these results are difficult to interpret and may simply reflect the differences in differentiation states of the normal keratinocytes and the cervical carcinoma lines that were analyzed. 

The most compelling evidence for aberrant *HOX* gene expression in cervical carcinoma was provided by a study performed with clinical specimens. Gene expression analysis of normal cervix, high-grade premalignant lesions and frank cancers provided strong evidence for upregulation of *HOXC10* expression during cervical cancer progression. Using cell culture based experiments the authors were able to link *HOXC10* expression to the acquisition of the invasive phenotype during cervical carcinogenesis [29].

## 3. Association of HPV16 E7 with Polycomb Group Proteins

### 3.1. HPV16 E7

The HPV16 E7 ORF encodes a low-molecular size, 98 amino acid protein that lacks intrinsic enzymatic and specific DNA binding activities. E7 is consistently expressed in cervical cancer even after integration of the viral genome into a host chromosome. It exerts its biological activities through associating with and functionally reprogramming host protein complexes that play critical roles in signal transduction. The best-studied cellular target of HPV16 E7 is pRB, the protein encoded by the retinoblastoma susceptibility gene (RB1), and the related p107 (RBL1) and p130 (RBL2) proteins [11]. However, additional important cellular targets of HPV16 E7 exist, and with the development of proteomic methods, identification of putative transformation targets for the HPV E7 protein has been a mainstay of HPV research [30]. 

### 3.2. Association of HPV16 E7 with E2F6 Containing Polycomb Repressive Complexes

One of the first proteomic studies with twin-epitope tagged HPV16 E7 ectopically expressed in HeLa cells, led to the identification of multiple previously unappreciated cellular HPV16 E7 targets, including the E2F6 transcriptional repressor [31,32]. E2F6 is a non-canonical member of the E2F family of transcription factors as it lacks the C-terminal binding site for pRB family member, and hence its transcriptional activity is not modulated by pRB binding. Like other E2F family members, E2F6 associates with a dimerization partner (DP1 or DP2) to form a transcriptionally active DNA binding complex. The E2F6/DP1 or DP2 heterodimer functions as a transcriptional repressor that is expressed in late S-phase, presumably to down-regulate E2F transcriptional target genes and stimulate S-phase exit [33,34]. HPV16 E7 associates with the C-terminal repressor domain of E2F6 and abrogates its repressive activity on E2F6 target genes. The ability to target E2F6 is shared with low-risk HPV E7 proteins, and the simian vacuolating virus 40 large tumor antigen (SV40 TAg) and the adenovirus (Ad) E1A protein also share the ability to inhibit E2F6 repression [32].

E2F6 is also a component of PRCs and several, apparently distinct, E2F6 containing PRCs have been described [35,36,37,38]. Examination of E7 associated proteins revealed evidence for the presence multiple additional PRC components, including BMI1, PCGF2 (MEL-18), CBX4 (hPC2), RING1, MGA and L3MBTL2 [32]. Many of these proteins have been specifically described as components of E2F6-associated PRCs, and hence it is likely that HPV16 E7 can associate with and potentially modify activities of E2F6 containing PRCs. It has not been investigated whether low-risk HPV E7 proteins, SV40 TAg or Ad E1A can also associate with E2F6 containing PRCs. 

Detection of E2F6 containing nuclear dots, presumably representing E2F6 containing PRCs were diminished in HPV16 E7 expressing cells, and an HPV16 E7 mutant that does not associate with E2F6 had no effect on the number of detectable E2F6 nuclear dots [32]. This finding is consistent with the model that E7 binding may alter the composition of E2F6 containing PRCs. This model, however, has not yet been tested experimentally.

### 3.3. Decreased Trimethylation of Lysine 27 of Histone H3 in HPV16 E7 Expressing Cells

Analysis of H3K27me3 levels in HPV16 E7 expressing primary human keratinocytes revealed a dramatic decrease in the H3K27me3 mark compared to donor and passage matched control vector expressing keratinocytes. Interestingly, there was no comparable decrease in the H3K27me2 and H3K27me1 marks [39]. Similar decreases in the H3K27me3 mark were also detected in clinical specimens of HPV16 positive high-grade premalignant squamous intraepithelial lesions (SILs) of the cervix [39,40]. Studies with a U2OS human osteosarcoma cell line with tetracycline-inducible E7 expression showed that the observed decrease of the repressive H3K27me3 mark is a direct and immediate effect of HPV16 E7 expression [39]. H3K27 staining diminishes within 72 hours of E7 induction and is restored when E7 expression is switched off. These results may provide an alternative explanation for the observed loss of detection of E2F6 containing PRCs in HPV16 E7 expressing cells.

## 4. Modulation of Polycomb Group Protein Expression by HPV16 Oncoproteins

### 4.1. Increased EZH2 Expression in HPV E7 Expressing Cells

The enzymatic PRC2 component, the H3K27 methyltransferase EZH2, is a bona fide human oncogene that is overexpressed and amplified in human tumors [41,42,43]. EZH2 transcription is regulated by E2F transcription factors [41]. HPV16 E7 targets pRB, p107 and p130 for proteasomal degradation and associates with E2F6 and therefore interferes with E2F transcriptional repressor activities, causing deregulated and increased expression of E2F transcriptional targets. Consistent with this model, HPV16 E7 transcriptionally activates EZH2 through its E2F sites and EZH2 is highly overexpressed in cervical lesions and tumors [44]. Interestingly, cervical carcinoma cells appear addicted to EZH2; depletion caused G1 cell cycle arrest and a low level of apoptosis.

High levels of EZH2 expression in HPV-positive cervical carcinomas and HPV E7 expressing cell lines and their apparent addiction to EZH2 is particularly remarkable since the H3K27me3 mark is decreased, not increased, in such cell lines. Different potential mechanistic explanations have been proposed to account for this apparently paradoxical finding. There is evidence that the enzymatic activity of EZH2 in PRC2 complexes is negatively regulated by AKT mediated phosphorylation at serine residue (S) 21 [45]. Since HPV16 E6 and E7 have both been reported to cause AKT activation [46,47] it is conceivable that PRC2 complex-associated EZH2 enzymatic activity may be low despite high-level overexpression in HPV16 positive lesions and cancers. A recent exciting study has shown that EZH2 phosphorylation on S21 acts as a functional switch that promotes EZH2 association with an androgen receptor containing transcription factor complex and in this context EZH2 acts as a polycomb independent activator of gene expression [48]. This study also provided compelling evidence that the oncogenic activity of EZH2 in castration-resistant prostate cancer cells is based on this polycomb independent activity of EZH2 [48]. EZH2 has also been detected in PRC complexes that catalyze H1K26 methylation [49]. It will be important to determine the posttranslational modifications of EZH2 and the biochemical composition of EZH2 complexes in HPV16 E7 expressing cells.

### 4.2. Modulation of BMI1 Expression by HPV E6 and E7

Expression of other PcG proteins in HPV16 E6/E7 expressing cells has also been studied. Levels of the PRC1 components SUZ12 and EED were reported to be unchanged but the levels of BMI1 were dramatically reduced in HPV16 E6/E7 expressing cells [40]. These results were somewhat surprising since BMI1 is a well-known oncogene that is frequently overexpressed in human tumors including cervical carcinomas [50,51,52,53], and it has even been suggested that BMI1 autoantibodies may be useful as a biomarker of cervical carcinomas [54]. 

In another study, HPV16 E6 was shown to increase BMI1 expression in primary human keratinocytes, and levels remained high in HPV E6/E7 immortalized keratinocyte lines. Most intriguingly, BMI1 could substitute for E6 and cooperate with E7 to cause keratinocyte immortalization [55]. This result is consistent with an earlier study that showed that BMI1 depletion in the HPV18 positive HeLa cervical carcinoma line caused S-phase depletion and G1 growth arrest [56].

## 5. Modulation of PRC Regulators by HPV16 E7

### 5.1. Upregulation of the H3K27 Demethylases KDM6A and KDM6B

**Figure 3:**
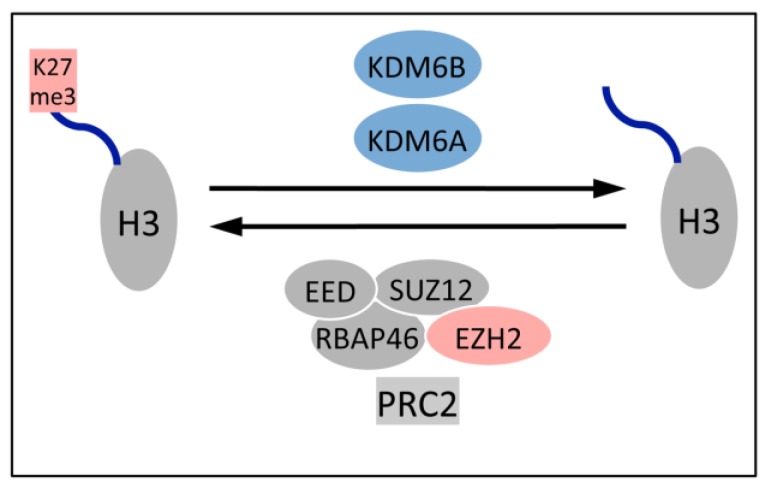
PRC2 mediated gene silencing is reversible. The EZH2 histone methyl transferase in PRC2 silences gene expression by adding the trimethyl mark on lysine residue 27 of histone H3, and this mark can be removed by one of the two H3K27 specific demethylases, KDM6A or KDM6B. See text for details.

H3K27 trimethylation is a dynamic process that is reversed by two demethylases, KDM6A (UTX) and KDM6B (JMJD3) (Figure 3). A mechanistic explanation for the dramatically decreased H3K27me3 levels in HPV16 positive cervical lesions and cancers was provided by the finding that KDM6A and KDM6B are expressed at markedly higher levels in these cells [39]. 

Even though KDM6A and KDM6B have identical catalytic activities and substrate specificities, they have different chromatin targets. KDM6A is encoded on the X-chromosome but escapes X-inactivation [57]. A related gene, UTY, is encoded on the Y chromosome but the UTY protein appears to lack histone demethylase enzymatic activity [58]. 

The fact that KDM6A and KDM6B have non-overlapping and non-redundant biological activities and that KDM6A may have biological activities that are independent of enzymatic activities have been impressively validated in genetically engineered mouse models (GEMMs). Studies with KDM6A^−/−^ GEMMs revealed that KDM6A plays an important role in activating cardiac differentiation [59]. KDM6A^−/−^ female mice died at E12.5 with defects in neural tube, yolk sac, and cardiac development, whereas approximately 20% of KDM6A^−/−^ male mice survived. These animals are fertile, but are smaller and have a reduced life span [58,60,61]. Studies with embryonic stem (ES) cells derived from these GEMMs and GEMMs expressing enzymatically inactive KDM6A, revealed that KDM6A regulated embryonic development may be independent of its enzymatic activity [61]. 

KDM6B^−/−^ GEMMs show perinatal lethality due to respiratory failure, which is caused by a developmental defect of the respiratory neuronal network [62]. KDM6B may also have biological activities that may not dependent on H3K27 demethylation. In macrophages KDM6B is induced by NF-kB in response to inflammatory stimuli and modulates expression of PRC silenced genes [63]. This KDM6B activity also does not appear to primarily depend on H3K27 demethylation [64]. 

The observed non-redundant activities of KDM6A and KDM6B may reflect incorporation of the two proteins into different multiprotein complexes. KDM6A is associated with H3K4 methyl transferase activity containing Mixed-Lineage-Leukemia (MLL)2/3 complexes [65,66]. KDM6A containing MLL2/3 complexes might link H3K27 demethylation to H3K4 methylation, and KDM6A/MLL2/3 complexes can convert transcriptionally silenced chromatin to a transcriptionally competent state. KDM6A mutations have been detected in some tumors [67], whereas KDM6B is upregulated in some cancers, particularly metastatic prostate carcinomas [68]. 

HPV16 E7 upregulates expression of KDM6A and KDM6B, at least in part at the level of transcription. The exact mechanisms of transcriptional induction of KDM6A and KDM6B expression remain to be determined. Upregulation of KDM6B, however, is clearly independent of HPV16 E7-mediated pRB degradation and activation of canonical E2F transcription factors [39].

### 5.2. KDM6B Controls Epithelial Differentiation

**Figure 4:**
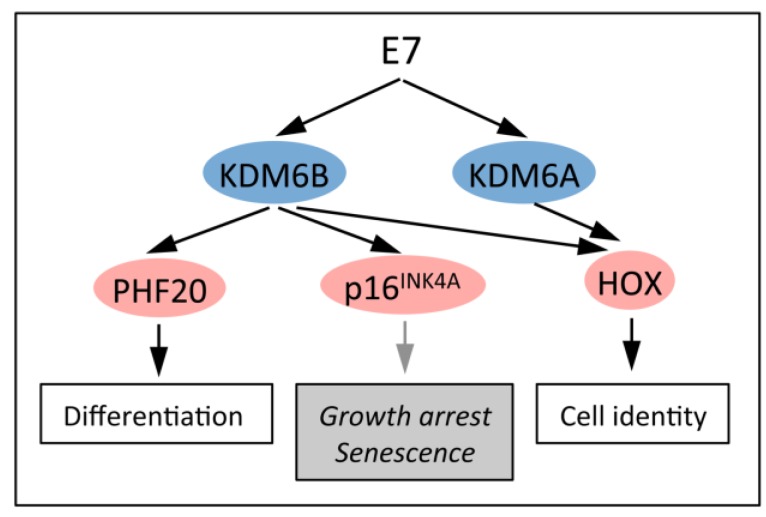
HPV16 E7 causes increased KDM6A and KDM6B expression. E7 mediated KDM6A and KDM6B induction affects multiple transcriptional programs, including keratinocyte differentiation and cell identity. KDM6B induction represents a cellular defense response to E7 oncogene expression that is to trigger cell cycle arrest and senescence. HPV16 E7 short-circuits this response by targeting the retinoblastoma tumor suppressor for degradation. Several other viruses including EBV have developed strategies to subvert such cellular defense responses. See text for details.

Genes that are specifically expressed during skin differentiation frequently carry H3K27me3 repressive marks in undifferentiated basal epithelial cells and are subject to PRC transcriptional regulation. Differentiation causes removal of these marks and relief from PCG repression. Even though KDM6B levels remain unchanged during differentiation, the removal of the repressive H3K27me3 mark is mostly mediated by KDM6B. This is based on the observation that KDM6B depletion in keratinocytes inhibited differentiation whereas ectopic expression accelerated differentiation. KDM6B enzymatic activity was required for acceleration of epithelial differentiation [69]. These results predict that HPV16 E7 expression not only changes *HOX* gene expression but also induces expression of genes that are normally only expressed in differentiating epithelial cells (Figure 4). This might play an important role in the viral life cycle as the switch from early to late viral gene expression is mediated by differentiation cues.

### 5.3. KDM6B Mediates the Oncogene-Induced Senescence (OIS) Response to RAS/RAF

**Figure 5:**
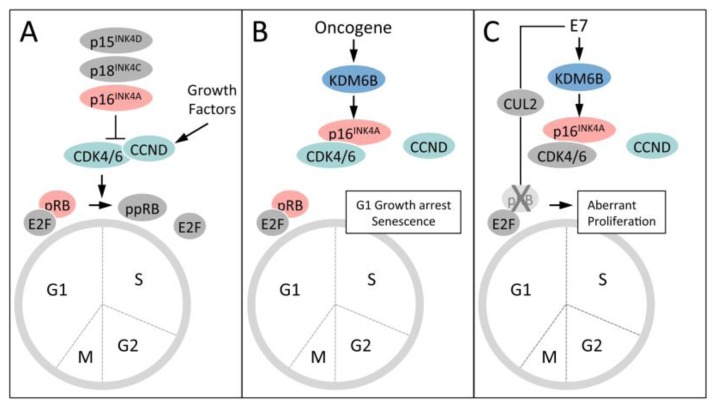
Induction and abrogation of the Oncogene Induced Senescence (OIS) tumor suppressor response by HPV16 E7. (**A**) The retinoblastoma tumor suppressor (pRB) pathway regulates S-phase cell cycle entry. Activity of pRB is regulated by phosphorylation by cyclin dependent kinase 4 or 6 (CDK4/6). CDK4/6 activity is activated by association with a D-type cyclin (CCND) and negatively regulated by association with one of several small molecule inhibitors (p16^INK4A^, p18^INK4C^ and p19^INK4D^), which are induced in response to different growth suppressive stimuli. CDK4/6 phosphorylation of pRB at the G1/S boundary inactivates the transcriptional repressor activity of the pRB/E2F complex and signals S-phase entry. (**B**) Oncogenic stimuli such as RAS/RAF signaling or HPV16 E7 expression causes KDM6B expression and de-repression of p16^INK4A^ expression, which inhibits CDK4/6 activity and pRB phosphorylation, causing G1 cell cycle arrest and senescence (OIS). (**C**) HPV16 E7 has evolved to evade OIS by targeting pRB for ubiquitin mediated proteasomal degradation.

Oncogene induced senescence (OIS) is one of the cell intrinsic tumor suppressor responses that has evolved to eliminate aberrantly proliferating and therefore potentially tumorigenic cells from the proliferative pool. OIS was initially discovered with the *RAS* oncogene and is signaled through transcriptional upregulation of the p16^INK4A^ tumor suppressor [70] (Figure 5). In some cell types, particularly in mouse cells, the partially overlapping gene encoding p14^ARF^ (or p19^ARF^ in mouse cells) is also induced and causes activation of the p53 tumor suppressor by inhibiting ubiquitination by MDM2 [71]. The p16^INK4A^ protein is a component of the retinoblastoma tumor suppressor pathway and inhibits CDK4/6-cyclin D mediated pRB phosphorylation and S-phase entry (Figure 5). It is frequently mutated, deleted or transcriptionally silenced in human tumors and tumor cell lines, supporting the notion that overcoming the OIS response is a major bottleneck in human cancer development.

Expression of p16^INK4A^ in normal human cells is generally low because the gene is silenced by H3K27 trimethylation and PRCs. In response to RAS/RAF stimulation, KDM6B expression is transcriptionally activated, potentially through AP1, causing removal of the H3K27me3 mark and enhanced p16^INK4A^ expression. This then signals G1 arrest and senescence through activation of the pRB tumor suppressor. Notably, however, RAS/RAF stimulation does not induce KDM6A expression nor does it consistently cause de-silencing of the p14^ARF^ promoter [71,72].

### 5.4. HPV16 E7 Induces the Cervical Cancer Biomarker p16^INK4A^ through KDM6B

The p16^INK4A^ tumor suppressor is an important biomarker for high-risk HPV-associated lesions and cancers and is induced by E7 [73,74]. It was initially thought that E7 induced p16^INK4A^ through E2F activation [75], but there are no E2F response elements in the p16^INK4A^ promoter and a pRB binding/degradation deficient HPV16 E7 mutant that does not activate E2F transcription, induces p16^INK4A^ expression as efficiently as wild-type HPV16 E7 [39]. More recent studies have clearly shown that high level p16^INK4A ^expression in cervical lesions and cancer represents a readout of an E7 induced OIS response [39]. How E7 triggers the response and how it is signaled to the KDM6B promoter is unclear, but E7 proteins also blunts OIS signaling by targeting pRB for ubiquitin dependent proteasomal degradation. Indeed, earlier studies have shown that pRB degradation, not merely binding, is necessary to fully inhibit pRB induced senescence [76]. Notably, p14^ARF^ expression is also increased in E7 expressing cells but in contrast to p16^INK4A^, depletion of KDM6B does not decrease p14^ARF^ levels or the H3K27me3 mark at the p14^ARF^ promoter [39].

Abrogation of the p16^INK4A^ OIS block in primary lymphocytes also represents a major roadblock to transformation by Epstein-Barr virus (EBV) [77]. The EBV proteins EBNA3A and EBNA3C cooperate to epigenetically silence the p16^INK4A^ promoter through a mechanism that involves the transcriptional repressor C-terminal binding protein (CtBP) [78,79]. Transcriptional silencing of other PRC controlled genes, such as the apoptosis regulator BIM, likely also contribute to EBV transformation [80]. Similarly, the hepatitis B virus (HBV) X protein downregulates the PRC2 protein SUZ12, which is essential for placing the H3K27me3 mark [81].

It is interesting to note that high-level p16^INK4A^ expression has also been detected in a number of other cancer types, including lung, prostate, breast and ovarian cancers [82,83,84,85]. In most cases, high level p16^INK4A^ expression is linked to pRB mutations [86], and it will be important to determine whether p16^INK4A^ expression is also mediated by KDM6B in such tumors. As mentioned previously, KDM6B upregulation has been reported in metastatic prostate carcinomas [68].

Cervical carcinoma cells are “addicted” to HPV E6/E7 oncogene expression and undergo growth arrest, senescence and cell death when expression of the viral oncoproteins is silenced [13,14,15]. Loss of HPV E6 or E7 expression can each individually induce senescence through re-activation of the p53 and pRB pathways, respectively [87,88,89]. It will be interesting to determine whether epigenetic mechanisms in general and PcG group proteins in particular are involved in signaling induction of senescence responses in these cases.

### 5.5. Dysregulated Homeobox Gene Expression in HPV16 E7 Expressing Cells

In contrast to RAF/RAS stimulation, HPV16 E7 also causes increased expression of the H3K27-specific demethylase KDM6A [39,40]. It is not clear whether KDM6A expression also represents a cellular tumor suppressive defense response, how E7 triggers it and what the transcriptional consequences of KDM6A expression may be. The fact that KDM6A has been classified as a putative tumor suppressor [67] may support the model that KDM6A may also be induced as a consequence of E7 triggering a cell intrinsic tumor suppressive defense response. Therefore it will be important to determine the mechanisms of E7 mediated KDM6A induction, the transcriptional consequences of KDM6A expression and the mechanisms by which E7 subverts KDM6A tumor suppressor activity.

It is known that KDM6A controls expression of *HOX* genes and indeed, *HOX* gene expression is dysregulated in HPV16 E7 expressing cells [39]. As pointed out in a previous section, *HOX* gene expression is frequently dysregulated in human cancers, including cervical carcinomas, and some *HOX* genes have been shown to contribute to cancer formation. Aberrant *HOX* gene expression in E7 expressing cells suggests that *HOX* gene dysregulation observed in cervical carcinoma cells is caused by HPV E7 expression and suggests that HPV infection causes reprogramming of the epigenetic make up of epithelial cells.

### 5.6. KDM6A and KDM6B are Essential in Cervical Carcinoma Cells

It is not intuitively obvious that KDM6A and/or KDM6B upregulation by HPV16 E7 would contribute to E7 transformation, since abrogation of downstream transcriptional consequences, such as p16^INK4A^ mediated OIS signaling, is critical for HPV, EBV and possibly HBV cell transformation. Hence, it was surprising that KDM6A as well as KDM6B depletion dramatically inhibited viability of the HPV16 positive CaSki cervical carcinoma line [39]. This finding suggests that survival of HPV E7 expressing cells is critically dependent on KDM6A and KDM6B targets. Even though it cannot be ruled out that these enzymes may have substrates other than methylated H3K27, it is likely that these represent transcriptional targets. The full delineation of KDM6A and KDM6B targets represents a major effort, but it is feasible by determining the full spectrum of human genes that have altered H3K27me3 marks in response to KDM6A or KDM6B using chromatin immunoprecipitation followed by DNA sequencing (ChIPSeq) in combination with expression profiling.

## 6. Concluding Remarks

Dysregulation of the histone code and activation of transcriptional programs in the “wrong” cell type is a hallmark of carcinogenesis. The REST transcriptional repressor, for example, is responsible for silencing neuronal-specific gene expression in non-neuronal cells. REST is a major tumor suppressor in colon, lung and breast cancers [90,91,92] and may be key to the neuro-endocrine character of some of these tumors. Similarly, PcG group proteins have been implicated in human carcinogenesis and some, including *BMI1* and *EZH2* are bona fide oncogenes that are overexpressed in many tumor types. The H3K27 demethylases KDM6A and KDM6B have also been implicated in carcinogenesis and the finding that KDM6B is the major mediator of OIS further supports the involvement of these enzymes in cancer formation.

One of the most exciting possibilities is that, unlike genetic mutations, epigenetic alterations may be reversed by inhibiting the enzymes that cause them. Even though some functions of KDM6A and KDM6B appear to be independent of their enzymatic activities, a recently developed KDM6 selective inhibitor was show to affect proinflammatory signaling in macrophages [93], even though previous studies suggested that KDM6B modulation of expression of PRC silenced genes in response to inflammatory stimuli [63] may not primarily depend on H3K27 demethylation [64]. Given the result that KDM6A as well as KDM6B expression is essential for viability to cervical carcinoma cells, KDM6 inhibition should be evaluated as a therapeutic modality for some human cancers. 
